# Resveratrol prevents age‐related heart impairment through inhibiting the Notch/NF‐κB pathway

**DOI:** 10.1002/fsn3.3817

**Published:** 2023-11-13

**Authors:** Le‐Feng Wang, Wen‐Juan Li, Xian‐Yi Zhang, Yi‐Chi Zhang, Guang‐Feng Chen, Xing‐Yu Zhou, Dong‐Mei Xv, Qiong Wu

**Affiliations:** ^1^ Jiangxi Province Key Laboratory of Laboratory Medicine Department of Clinical Laboratory The Second Affiliated Hospital of Nanchang University Nanchang China; ^2^ Department of Ophthalmology West China Hospital Sichuan University Chengdu China; ^3^ State Key Laboratory of Food Science and Technology Nanchang University Nanchang China

**Keywords:** aging, heart impairment, inflammation, Notch/NF‐κB pathways, oxidative stress, resveratrol

## Abstract

Resveratrol (RSV) is a natural polyphenol compound found in various plants that has been shown to have potential benefits for preventing aging and supporting cardiovascular health. However, the specific signal pathway by which RSV protects the aging heart is not yet well understood. This study aimed to explore the protective effects of RSV against age‐related heart injury and investigate the underlying mechanisms using a D‐galactose‐induced aging model. The results of the study indicated that RSV provided protection against age‐related heart impairment in mice. This was evidenced by the reduction of cardiac histopathological changes as well as the attenuation of apoptosis. RSV‐induced cardioprotection was linked to a significant increase in antioxidant activity and mitochondrial transmembrane potential, as well as a reduction in oxidative damage. Additionally, RSV inhibited the production of pro‐inflammatory cytokines such as interleukin‐1β (IL‐1β) and tumor necrosis factor‐α (TNF‐α). Furthermore, the expression of toll‐like receptor 4 (TLR4), nuclear factor kappa‐B p65 (NF‐κB p65), and notch 1 protein were inhibited by RSV, indicating that inhibiting the Notch/NF‐κB pathway played a critical role in RSV‐triggered heart protection in aging mice. Moreover, further data on intestinal function demonstrated that RSV significantly increased short‐chain fatty acids (SCFAs) in intestinal contents and reduced the pH value in the feces of aging mice. RSV alleviated aging‐induced cardiac dysfunction through the suppression of oxidative stress and inflammation via the Notch/NF‐κB pathway in heart tissue. Furthermore, this therapeutic effect was found to be associated with its protective roles in the intestine.

## Graphical abstract

Resveratrol alleviates the D‐gal‐induced cardiac aging process. Resveratrol alleviates mayocardial injury by blocking the Notch/NF‐κB pathway. Resveratrol could significantly improve short‐chain fatty acids in the feces of mice.

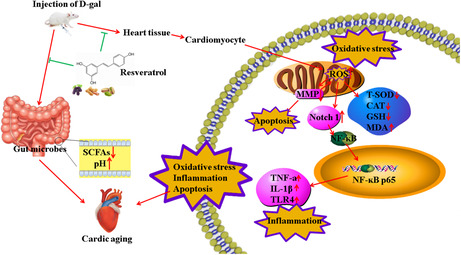

## 1 INTRODUCTION

Aging is a complex process of degeneration that increases the risk of various chronic diseases (Kazemi Pordanjani et al., 2023). Cardiovascular disease (CVD) represents a major complication of aging and is recognized as the main cause of mortality among elderly individuals. The oxidative stress theory is currently considered the most reasonable mechanism of aging related‐damage (Salemi et al., 2023). The excessive production of reactive oxygen species (ROS) could result in oxidative stress and pro‐inflammatory responses, causing protein oxidation, lipid peroxidation, and DNA damage. Therefore, ROS could affect normal cell function and even lead to apoptosis (Wang et al., 2021). Fortunately, the anti‐oxidant agent can remove ROS, which slows down aging and reduces the risk of relevant diseases. As a result, some natural antioxidants were used to prevent damage caused by oxidative stress in some age‐related diseases (Chen et al., 2023).

Resveratrol (RSV), a plant‐derived polyphenol that is found in human foods such as peanuts, grapes, red wine, and berries, has attracted considerable attention due to its beneficial role in preventing aging by repairing the redox system (Koushki et al., 2018). In addition to its anti‐aging capacities, RSV has also been shown to possess protective effects against CVD by scavenging ROS and promoting the activities of various antioxidant enzymes (Carrizzo et al., 2013). Apart from the oxidative stress theory, inflammation has been strongly implicated in the pathogenesis of aging. In recent decades, accumulating studies have revealed that inflammation plays a critical role in causing age‐related diseases, including cardiovascular diseases (Alemany et al., 2014; Steven et al., 2019). It has been suggested that the nuclear factor kappa‐B p65 (NF‐κB p65) signaling pathway is involved in the occurrence and development of aging and its complications (Kanigur Sultuybek et al., 2019; Salminen & Kaarniranta, 2009). Increasing investigations have supported that Notch was an imperative upstream element to trigger NF‐κB. Notch expression was upregulated in response to aberrant inflammation and then affected the subsequent NF‐κB pathway (Xiao et al., 2018). In recent years, scientific investigations have demonstrated the advantages of short‐chain fatty acids (SCFAs) for human health. These SCFAs are classified as diminutive organic monocarboxylic acids possessing carbon chain lengths ranging from two to six (Tain et al., 2021). SCFAs possess the ability to mitigate systemic inflammatory and metabolic illnesses (Li et al., 2020). Currently, there is limited evidence available to support the notion that RSV can prevent age‐related heart dysfunction via the blockade of the Notch/NF‐κB pathway. Additionally, in a D‐galactose (D‐gal)‐induced aging model, it is currently unknown if RSV‐induced heart protection is associated with SCFAs.

Therefore, we hypothesized that RSV mediated its cardioprotective effects by regulating the Notch/NF‐κB pathway and SCFAs. In animal studies, D‐gal was a common modeling drug used to simulate the natural aging process (Qian et al., 2021). It can induce metabolic disturbances in cells, alter oxidative enzyme activity, increase superoxide anion and oxidative product production, and cause damage to proteins, lipids, and DNA. Furthermore, animal aging models generated by D‐gal can simulate the normal aging process (El‐Far et al., 2021). This resulted in apoptosis and inflammation, which ultimately contributed to the aging process. Therefore, this study aimed to investigate whether RSV could protect against D‐gal‐induced cardiac aging and elucidate its underlying mechanisms using various parameters related to oxidative stress, inflammation, the Notch/NF‐κB pathway, and SCFAs.

## 2 MATERIALS AND METHODS

### 2.1 Reagents

D‐gal and RSV were purchased from Sigma. Hematoxylin and eosin (H&E) solution was provided by Servicebio Biotech. The Annexin V‐FITC apoptosis detection kit was purchased from BD Biosciences. Rhodamine 123 (Rho 123) and 2′,7′‐dichlorofluorescein diacetate (DCFH‐DA) were provided by Molecular Probes Inc. Interleukin‐1β (IL‐1β) and tumor necrosis factor‐α (TNF‐α) enzyme‐linked immunosorbent assay (ELISA) kits were obtained from Boster Biology Engineering Institute. Total superoxide dismutase (T‐SOD) kit, catalase (CAT) kit, malondialdehyde (MDA) kit, and glutathione (GSH) kit were obtained from Nanjing Jiancheng Bioengineering Institute. A protein isolation kit was procured from Kaiji Biological Co., Ltd. Anti‐Notch1, anti‐Toll‐like receptor 4 (TLR4), anti‐NF‐κB, anti‐Histone H2A.X, and anti‐β‐actin primary antibodies, as well as the corresponding secondary antibodies, were purchased from Abcam. A 10% neutral formalin fixative solution was supplied by Solaibao Technology Co., Ltd.

### 2.2 Animals

A total of 60 female/male BALB/c Kunming mice aged 6–8 weeks (cleaning grade), with an average weight of 30.00 ± 2.00 g, were procured from Jiangxi University of Traditional Chinese Medicine in Jiangxi, China [Certificate Number SCXK (gan) 2018‐0003]. The mice were housed in groups in an animal room under controlled temperature (18–22°C) and relative humidity (50%–60%) and maintained under standard laboratory conditions with the natural dark and light cycles. They were allowed ad libitum access to tap water and food, and the litter, feed, and drinking water all met the relevant use standards. The guidelines for the Care and Use of Laboratory Animals (NIH Publication No. 85‐23, revised 1996) were followed during all the experiments. All procedures described were reviewed and approved by the Animal Care Review Committee (approval number 0064257) at Nanchang University, China.

### 2.3 Experimental protocols

Mice were randomly divided into five groups (*n* = 12): the control group, the D‐gal group, the D‐gal+RSV‐low‐dose group (RSVL, 6.25 mg/kg), the D‐gal+RSV‐medium‐dose group (RSVM, 12.5 mg/kg), and the D‐gal+RSV‐high‐dose group (RSVH, 25 mg/kg). All of the groups except the control group were intraperitoneally injected with D‐gal at a dose of 100 mg/kg of body weight (BW) daily for 10 weeks. Starting from the seventh week, we dissolved RSV in physiological saline and administered it to three RSV‐treated groups by intragastric administration. The doses used were 6.25, 12.5, and 25 mg/kg. The remaining two groups received the same volume of normal saline in a similar manner. At the end of the 10th week, on the 70th day, all the mice were euthanized by cervical dislocation.

### 2.4 H&E staining

Hearts were harvested for H&E staining analysis and fixed in a 10% formalin solution for 24 h. The obtained tissues were further embedded in paraffin, then cut into slices, and stained with hematoxylin–eosin. Subsequently, the sections of tissue were observed under a light microscope (Nikon). They were then photographed and analyzed.

### 2.5 Determination of myocardial apoptosis

Heart tissues were cut into pieces, and the tissue fragments were incubated with 250 μL of 1.25 mg/mL trypsinase at room temperature for 10 min. Digestion was terminated with Dulbecco's Modified Eagle Medium (DMEM), and the digestive fluid was filtered and placed in a centrifuge tube, which was then centrifuged at 2000 r/min for 5 min. After discarding the culture supernatant, the precipitate was resuspended to yield cardiomyocytes and washed twice with PBS. Subsequently, Annexin V‐FITC (AV) staining and propidium iodide (PI) staining solutions were added to the cardiomyocytes, which were incubated on ice for 10 min and in the dark for an additional 5 min. The apoptosis rate of cardiomyocytes in each group was determined by flow cytometry (Becton Dickinson).

### 2.6 Determination of mitochondrial membrane potential (MMP) and ROS in cardiomyocytes

After digestion of heart tissue with collagenase II, the isolation of mouse cardiomyocytes was performed as described (Alam et al., 2020). Rho123 and DCFH‐DA were employed to determine MMP and ROS, respectively. According to the working principle, mitochondria could emit green fluorescence after uptaking Rho123 fluorescent dye, and the fluorescence intensity was positively correlated with MMP. DCFH‐DA was used as a ROS‐specific fluorescent probe to measure the level of ROS, and the fluorescence intensity of DCF was used to indicate the content of intracellular ROS, and the fluorescence intensity was proportional to ROS content (Zhou et al., 2017). In brief, we took the well‐grown cardiomycytes, incubated the cells with 10 mg/mL Rho123 or 20 μmol/L DCFH‐DA at 37°C for 20 min in the dark, removed them from the medium, and then rinsed the cells with PBS three times. Eventually, the sample was detected by flow cytometry (Becton Dickinson).

### 2.7 Determination of cardiac antioxidant enzyme activity and MDA content

10% homogenate of heart tissue was taken and devoted to measuring the content of T‐SOD, GSH, CAT, and MDA using commercial kits with a microplate reader (Thermo Multiskan MK3 Microplate Reader; Thermo Fisher Scientific), following the manufacturer's instructions (Huang et al., 2018).

### 2.8 Determination of cardiac cytokine secretion levels by ELISA

An appropriate amount of myocardial tissue was weighed and added to the pre‐cooled PBS buffer. Subsequently, the tissue was homogenized at a low temperature with a tissue homogenizer to obtain a 10% concentration of tissue homogenate. The supernatant was then centrifuged, divided, and stored at −80°C for sequential use. IL‐1β and TNF‐α levels were ultimately measured according to the corresponding ELISA kit instructions. The absorbance was measured at 570 nm by using a Multiskan MK3 Microplate reader (Thermo Fisher), and the cytokine concentrations were determined from the standard curve.

### 2.9 Western blot analysis

Heart tissue was harvested, homogenized, and then proteins were extracted using lysis buffer. Protein content was determined by the Bradford Protein Assay (Bio‐Rad Laboratories). Meanwhile, 12% or 15% separation gel and 5% concentrated gel were prepared in strict accordance with the standard procedure. Subsequently, protein samples were mixed with Laemmli sample buffer and boiled for 5 min. To ensure equivalent protein loading, 20 protein samples were added to each hole and electrophoreted for 15 min at 150 V and then at a voltage of 100 V for 60, 90, and 120 min. After incubation with 5% nonfat milk powder in TBST buffer for 1 h at room temperature, the membranes were immunoblotted overnight at 4°C with primary antibodies (1:1000 dilution). Then, detection of primary antibodies was performed using HRP‐conjugated secondary antibodies (1:3000 dilution). The resulting immune complexes were detected by ECL reagents and images analyzed by a Molecular Imager® system (Nikon). Meanwhile, we used the anti‐β‐actin antibody as an internal control.

### 2.10 Determination of colon contents, SCFAs, and pH in aging mice

100 mg of fecal samples were diluted in EP tubes at a ratio of 1:7 (w/v) with deionized water. Then, the tubes were exposed to an ultrasonic wave for 5 min and placed in an ice bath for 20 min. And samples were centrifuged at 4800 ***g*** for 20 min at 4°C. Finally, the supernatant was mixed for SCFA measurement using gas chromatography (GC, HP‐5MS capillary column, 30 m × 0.32 mm, 0.25 mm) analysis and pH measurement using a pH meter (Tianjing analytic corp.). The analytical conditions used by Agilent 6890N gas chromatography were based on the method of Hu et al. (2012).

### 2.11 Statistical analysis

Statistical analysis was performed using the IBM SPSS Statistics software, version 23.0. The mean values of different experimental groups were compared using one‐way ANOVA, followed by the Dunnett method for between‐group comparison. Data were expressed as mean ± SEM. *p* < .05 was regarded as statistically significant.

## 3 RESULTS

### 3.1 Effects of RSV on the cardiac histopathological changes of aging mice

**FIGURE 1:**
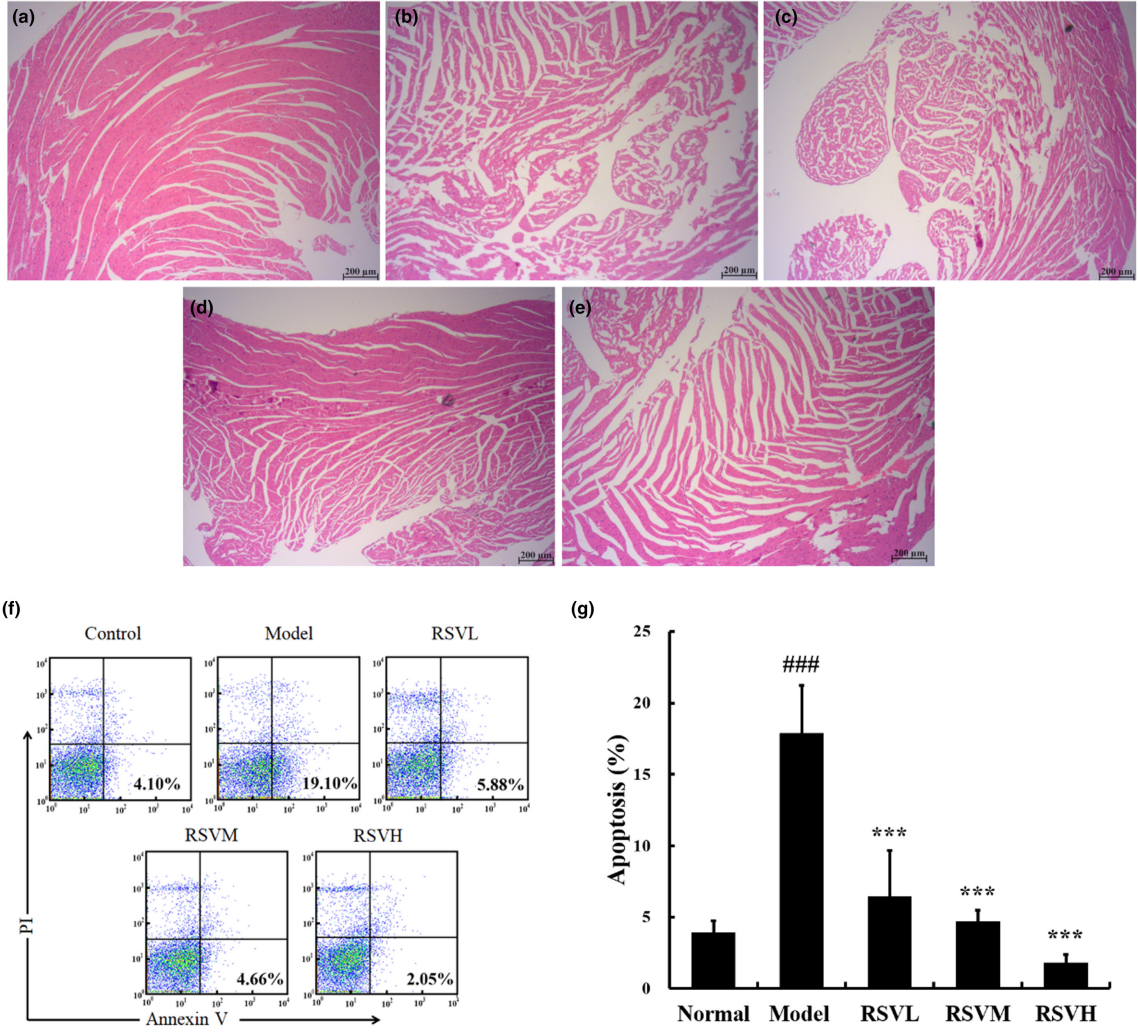
Pathological observation of heart tissue sections in mice and flow cytometric analysis of cardiomyocytes in aging mice. H&E staining showed that RSV protected against the structural damage caused by D‐gal, scale bar = 200 μm. (a) H&E stain of the cardiac tissue in the control group. (b) H&E stain of the cardiac tissue in the D‐gal group. (c) The RSVL group's cardiac tissues were stained with H&E. (d) The RSVM group's cardiac tissues were stained with H&E. (e) Cardiac tissues in the RSVH group were stained with H&E. (f) A flow cytometry assay showed that RSV attenuated apoptosis of cardiomyocytes in mice. (g) Quantitative analysis of cardiomyocyte apoptosis percentages in aging mice. Control group: treatment with normal saline; D‐gal group: 100 mg/kg, D‐gal‐treated alone; RSVL group (D‐gal+RSV‐low‐dose group): Treatment with D‐gal (100 mg/kg) and RSV (6.25 mg/kg); RSVM group (D‐gal+RSV‐medium‐dose group): Treatment with D‐gal (100 mg/kg) and RSV (12.5 mg/kg); RSVH group (D‐gal+RSV‐high‐dose group): Treatment with D‐gal (100 mg/kg) and RSV (25 mg/kg). Data are presented as mean ± SEM (*n* = 5 for each group). ^###^ *p* < .001 versus control group; ***p < .001 versus D‐gal group. (*n* = 5 for each group).

Figure 1a–e shows the cardiac tissue sections stained with H&E to observe the pathological structure in the current experiment. The control group (Figure 1a) exhibited a neat and intact heart structure, with well‐arranged myocardial fibers and normal cardiac structure. In contrast, myocardial fibers in the D‐gal group were obviously fractured, as shown in Figure 1b. The space between myofibrils was enlarged. Compared with the D‐gal group, the RSV treatment group exhibited significantly more regular arrangement of myocardial fibers, ameliorated fiber breakage, and the cardiac structure tended to be intact, as shown in Figure 1c–e. In conclusion, these results suggest that RSV could ameliorate swollen and vacuolated cardiomyocytes and the disorganization of myofibrils in the mouse heart.

### 3.2 Effects of RSV on apoptosis in myocardial cells

Apoptosis, the regulation of the homeostatic balance between cell proliferation and programmed cell death, is essential for the development and maintenance of multicellular organisms (Voss & Strasser, 2020). Excessive apoptosis may be equally harmful to the organism, indicating that tight regulation of the apoptotic machinery is essential for survival. Hence, to investigate the effect of RSV on myocardial injury in D‐gal‐induced aging mice, the apoptosis level of cardiomyocytes was evaluated. As shown in Figure 1f,g, the percentage of cardiomyocyte apoptosis increased from 3.95 ± 0.77% in the control group to 17.88 ± 3.34% in the D‐gal group, suggesting that D‐gal could enhance cardiomyocyte apoptosis. The percentage of cardiomyocyte apoptosis in the RSV‐treated groups was notably reduced, and the effect was elevated with the increase in RSV dosage (*p* < .001). In the RSVH group, the percentage of cardiomyocyte apoptosis was 1.84 ± 0.54%. These results demonstrated that RSV could attenuate the apoptosis of cardiomyocytes in aging mice and promote the survival of cardiomyocytes.

### 3.3 Effects of RSV on MMP and ROS generation in myocardial cells

**FIGURE 2:**
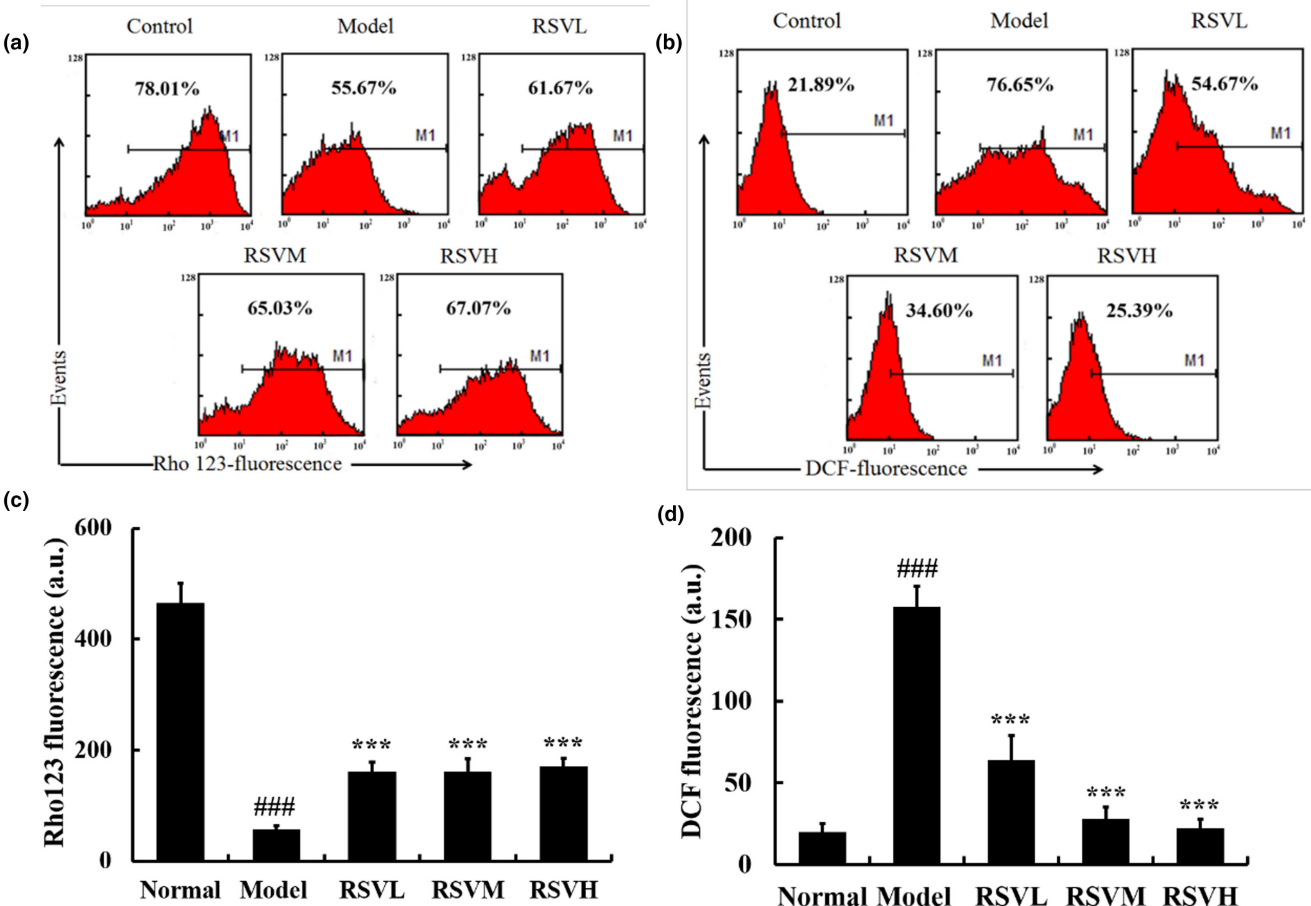
Flow cytometric analysis of mitochondrial membrane potential and ROS in aging mouse cardiomyocytes. (a) Flow cytometric analysis of mitochondrial membrane potential was performed with Rho123. RSV increased the levels of MMP in aging mice cardiomyocytes compared to the D‐gal group. (b) ROS distribution in aging mouse cardiomyocytes was measured by flow cytometry. Compared with the D‐gal group, RSV reduced the level of ROS in the cardiomyocytes of aging mice. (c) Quantitative analysis of the fluorescence intensity of Rho 123 in aging mice. (d) Quantitative analysis of the fluorescence intensity of DCF in aging mice. Control group: treatment with normal saline; D‐gal group: 100 mg/kg, D‐gal‐treated alone; RSVL group (D‐gal+RSV‐low‐dose group): Treatment with D‐gal (100 mg/kg) and RSV (6.25 mg/kg); RSVM group (D‐gal+RSV‐medium‐dose group): Treatment with D‐gal (100 mg/kg) and RSV (12.5 mg/kg); RSVH group (D‐gal+RSV‐high‐dose group): Treatment with D‐gal (100 mg/kg) and RSV (25 mg/kg). Data are presented as mean ± SEM (*n* = 5 for each group). ^###^ *p* < .001 versus control group; ***p < .001 versus D‐gal group.

Mitochondria were known to be largely implicated in apoptosis activation, and the collapse of the MMP was suggested to be an important early event of mitochondrial injury, leading to ROS overproduction and the release of apoptosis factors that finally induced apoptosis (Yi et al., 2022). After treatment with D‐gal for 10 weeks, low Rho 123 staining in cardiomyocytes reflected a markedly elevated loss of MMP when compared to the control group (*p* < .001; Figure 2a,c), and the collapse of MMP appeared in parallel with the enhancement of ROS generation (*p* < .001; Figure 2b,d). Treatment of D‐gal‐induced aging mice with RSV significantly alleviated the collapse of MMP and ROS enhancement compared with D‐gal treatment alone in mice (*p* < .001). These results indicated that RSV could potentiate MMP and inhibit ROS accumulation in myocardial cells in D‐gal‐induced aging mice, thereby protecting cardiomyocytes.

### 3.4 Effects of RSV on antioxidant activities and lipid oxidation in aging mouse hearts

**FIGURE 3:**
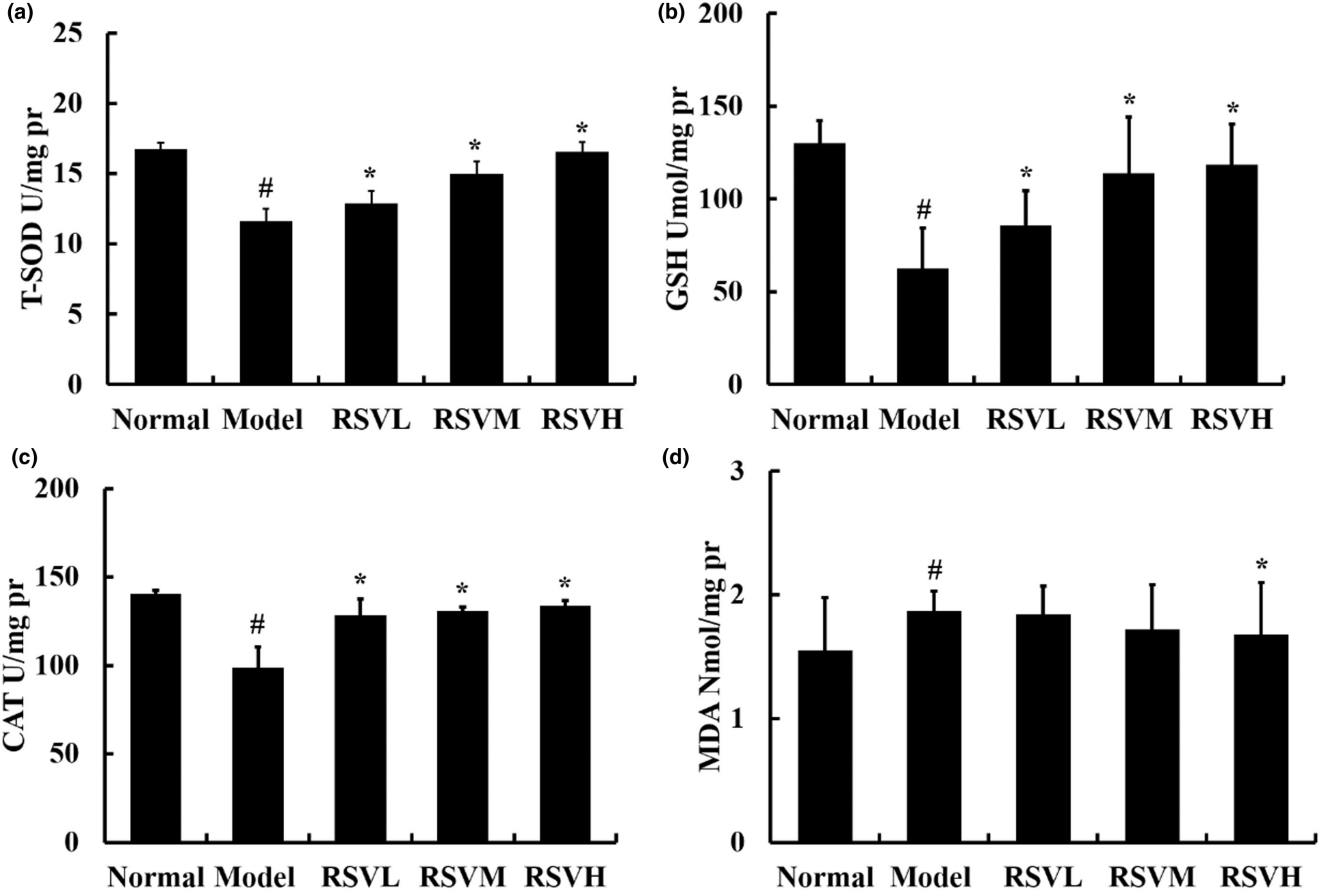
Cardiac antioxidant enzyme level and MDA content in mice. The activity of T‐SOD, CAT, and GSH was significantly increased in the RSV group compared with that in the D‐gal group. RSV significantly reduced MDA content when compared with D‐gal. (a) RSV's effects on T‐SOD in aging mouse hearts. (b) The effects of RSV on GSH in aging mouse hearts. (c) The effects of RSV on CAT in aging mouse hearts. (d) RSV's effects on MDA in aging mouse hearts. Control group: treatment with normal saline; D‐gal group: 100 mg/kg, D‐gal‐treated alone; RSVL group (D‐gal+RSV‐low‐dose group): Treatment with D‐gal (100 mg/kg) and RSV (6.25 mg/kg); RSVM group (D‐gal+RSV‐medium‐dose group): Treatment with D‐gal (100 mg/kg) and RSV (12.5 mg/kg); RSVH group (D‐gal+RSV‐high‐dose group): Treatment with D‐gal (100 mg/kg) and RSV (25 mg/kg). Data are presented as mean ± SEM (*n* = 5 for each group). ^#^ *p* < .05 versus control group; *p < .05 versus D‐gal group.

To investigate the role of RSV in antioxidant activities and lipid oxidation, T‐SOD, CAT, GSH, and MDA were measured in the subsequent experiments. As shown in Figure 3a, T‐SOD and CAT were suggested to be cellular antioxidant enzymes, and treatment of mice with D‐gal alone for 10 weeks significantly reduced the activities of T‐SOD and CAT when compared to the control group (*p* < .05). Similarly, GSH acted as a non‐enzymatic antioxidant, and the data showed that D‐gal‐treated mice exhibited a significant decrease in the D‐gal group. Meanwhile, MDA, an important product of lipid peroxidation, was significantly increased in the D‐gal group, confirming that oxidative stress was implicated in the pathogenesis of aging and CVD. Correspondingly, RSV inhibited the changes of T‐SOD, CAT, GSH, and MDA in D‐gal‐treated mice (*p* < .05), and these beneficial roles were most pronounced in the RSVH group. These findings indicated that RSV protected mice from age‐related heart impairment by modifying the redox system in vivo.

### 3.5 Effects of RSV on the expression of cytokines in aging mouse hearts

**FIGURE 4:**
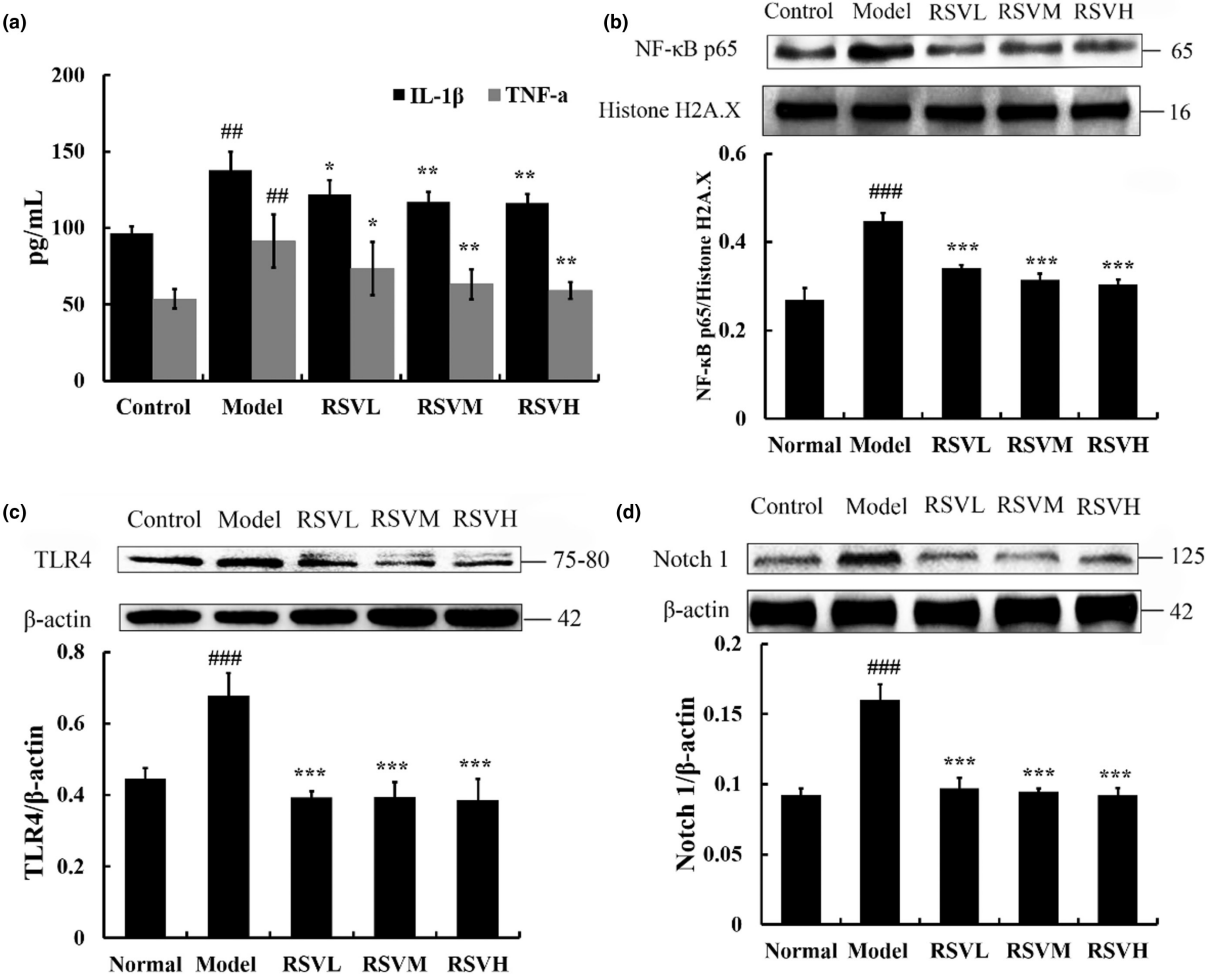
Effect of RSV on cytokine expression and Notch/NF‐κB pathway‐related protein expression in the heart tissue of mice. (a) Both IL‐1β and TNF‐α were significantly lower in the RSV‐treated group than in the D‐gal group. (b) When compared to the D‐gal group, RSV treatment reduced the expression of NF‐κB p65 substantially. (c) RSV treatment reduced TLR4 expression significantly compared to the D‐gal group. (d) RSV treatment significantly reduced Notch 1 expression compared to D‐gal treatment. Control group: treatment with normal saline; D‐gal group: 100 mg/kg, D‐gal‐treated alone; RSVL group (D‐gal+RSV‐low‐dose group): Treatment with D‐gal (100 mg/kg) and RSV (6.25 mg/kg); RSVM group (D‐gal+RSV‐medium‐dose group): Treatment with D‐gal (100 mg/kg) and RSV (12.5 mg/kg); RSVH group (D‐gal+RSV‐high‐dose group): Treatment with D‐gal (100 mg/kg) and RSV (25 mg/kg). Data are presented as mean ± SEM (*n* = 5 for each group). ^##^ *p* < .01 versus Control group; ^###^ *p* < .001 versus Control group; *p < .05 versus D‐gal group; **p < .01 versus D‐gal group; ***p < .001 versus D‐gal group.

IL‐1β plays a key role in immune response, autoinflammation, and tissue repair (Yi et al., 2022). TNF‐α is a pleiotropic cytokine that induces a variety of cellular responses, ranging from inflammatory cytokine production to cell survival, cell proliferation, and even cell death (Karki et al., 2021). As shown in Figure 4a the levels of IL‐1β and TNF‐α in the D‐gal group were significantly higher than those in the control group (*p* < .01). Compared with the D‐gal group, the concentrations of IL‐1β and TNF‐α were significantly reduced in the RSV‐treated group (RSVL group vs. D‐gal group, *p* < .05; RSVM and RSVH group vs. D‐gal group, *p* < .01). In the RSVH group, concentrations of proinflammatory cytokines IL‐1β and TNF‐α were 116.44 ± 5.7 pg/mL and 58.91 ± 5.45 pg/mL. The findings provided evidence that RSV inhibited the expression of IL‐1β and TNF‐α in the hearts of aging mice.

### 3.6 Effects of RSV on the expression of TLR4 and NF‐κB p65 proteins in aging mouse hearts

The NF‐κB pathway encompassed an important family of inducible transcription factors, which were critical for the regulation of gene expression in response to injury and inflammatory stimuli. Accordingly, TLR4 may propagate the NF‐κB pathway and facilitate the signal cascade, thereby contributing to the expression of inflammatory mediators and ultimately mediating myocardial injury (Qi et al., 2019). Thus, TLR4 and NF‐κB expression were investigated (Figure 4c). And the expression of TLR4 in the D‐gal group was higher than that of the control group (*p* < .001). Compared with the D‐gal group, the expression of TLR4 in the RSV‐treated group was diminished (*p* < .001), and these beneficial effects were presented in proportion to the dosage of RSV. In the RSVH group, the expression of TLR4 was 0.57 fold that of the D‐gal group, indicating that the expression of TLR4 was blocked dramatically in the hearts of aging mice. Furthermore, we also analyzed the content of NF‐κB p65 (Figure 4b). Likewise, the data showed that the expression of NF‐κB p65 was significantly boosted when compared to the control group (*p* < .001), and the change referring to the expression of NF‐κB p65 could be significantly subsided in the aging mice by the treatment of RSV (*p* < .001). Also, the expression of NF‐κB p65 was lowest in the RSVH group. Therefore, it was concluded that RSV possessed cardioprotective effects, which were implicated in the inhibition of the TLR4‐mediated NF‐κB pathway in aging mice.

### 3.7 Effects of RSV on the expression of Notch1 protein in aging mouse hearts

Notch signaling has recently attracted much attention due to its potential involvement in the onset and progression of CVD. It was believed to interact with the NF‐κB pathway, but the exact mechanisms are yet to be fully understood. In this study, the expression of Notch1 was found to be significantly higher in the D‐gal group compared to the control group (*p* < .001; Figure 4d). However, the expression of Notch1 was significantly reduced after treatment with RSV in the heart tissue of aging mice (*p* < .001). Our findings suggested that RSV‐mediated downregulation of Notch1 expression was associated with the inhibition of TLR4 and NF‐κB p65 proteins. These findings supported the crosstalk mechanisms between Notch and the NF‐κB pathway and indicated that cardioprotective effects were related to the Notch/NF‐κB pathway.

### 3.8 Effects of RSV on colon contents SCFAs and pH in a D‐gal‐induced cardiac aging mouse model

**FIGURE 5:**
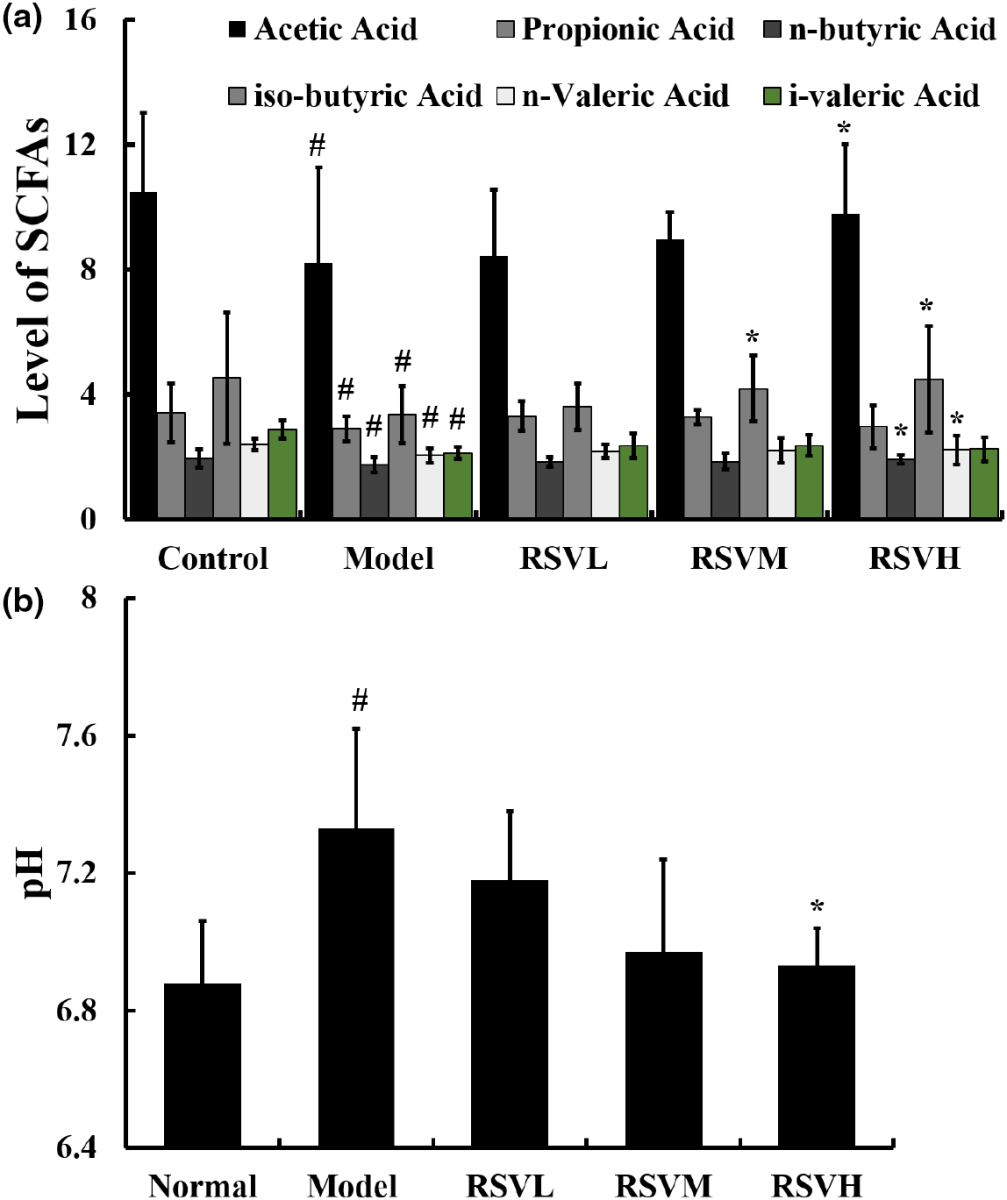
The effect of RSV on SCFA and pH in aging mice. (a) Secretion levels of SCFAs in the colonic contents of mice. RSV treatment increased secretion levels of acetic acid, propionic acid, n‐butyric acid, iso‐butyric acid, n‐valeric acid, and i‐valeric acid. (b) The pH of colon contents in mice. In comparison with the D‐gal group, the RSV group had a lower pH. Control group: treatment with normal saline; D‐gal group: 100 mg/kg, D‐gal‐treated alone; RSVL group (D‐gal+RSV‐low‐dose group): Treatment with D‐gal (100 mg/kg) and RSV (6.25 mg/kg); RSVM group (D‐gal+RSV‐medium‐dose group): Treatment with D‐gal (100 mg/kg) and RSV (12.5 mg/kg); RSVH group (D‐gal+RSV‐high‐dose group): Treatment with D‐gal (100 mg/kg) and RSV (25 mg/kg). Data are presented as mean ± SEM (*n* = 5 for each group). ^#^ *p* < .05 versus Control group; *p < .05 versus D‐gal group.

SCFAs serve as a link between the microbiota and host cells. As shown in Figure 5a, we investigated the role of colon contents, SCFAs, and pH in a D‐gal‐induced cardiac aging mouse model. The results showed a significant decrease in six SCFAs (acetic acid, propionic acid, n‐butyric acid, i‐butyric acid, n‐valeric acid, and i‐valeric acid) in the colon content of the D‐gal group compared to the control group (*p* < .05). In contrast, treatment of D‐gal‐induced aging mice with RSV demonstrated certain activities in recovering and increasing these SCFAs. Specifically, compared with the D‐gal group, treatment with RSVM and RSVH significantly enhanced iso‐butyric acid in the colon (*p* < .05). As for the other five SCFAs, RSV displayed a significant difference when compared to the D‐gal group. We subsequently investigated the impact of RSV on intestinal pH. As shown in Figure 5b, the pH in the D‐gal group was appreciably higher than that in the control group (*p* < .05). Compared with the D‐gal group, the RSV treatment group showed a decline in pH, among which the high‐dose group manifested the most evident decrease with statistical significance (*p* < .05). Collectively, these data showed that RSV could regulate SCFAs and pH in D‐gal‐induced cardiac aging mouse.

## 4 DISCUSSION

The incidence of cardiac dysfunction among the elderly is increasing, leading to a reduction in their quality of life and placing a huge burden on families and society (Zhang et al., 2019). RSV, a naturally occurring phytoalexin, has demonstrated various bioactivities, including antioxidant effects, anti‐aging effects, and cardioprotective effects (Xueyan et al., 2018). In this study, we confirmed the beneficial effects of RSV against D‐gal‐induced heart impairment in mice. Furthermore, we addressed the multiple mechanisms responsible for its cardioprotective effects, including its influence on oxidative stress, inflammatory, and apoptotic markers, as well as intestinal status.

ROS plays a significant role in the pathogenesis of aging in mammalian cells, and many age‐related chronic diseases, such as CVD, may result from ROS damage to biological macromolecules such as lipids, proteins, or DNA (Dubois‐Deruy et al., 2020). To eliminate ROS, the body is equipped with SOD, CAT, and GSH (Yi et al., 2020). Typically, MDA serves as a biomarker for ROS‐induced lipid peroxidation (Razi & Malekinejad, 2015). Apoptosis is largely induced by the accumulation of ROS, which damages DNA and leads to a collapse of MMP and cytochrome c leakage, triggering caspase activation (Kim et al., 2020). In our study, we observed increased levels of celluar antioxidant (GSH) and antioxidant enzymes T‐SOD and CAT in the heart tissues of aging mice treated with RSV. Additionally, MDA was significantly reduced in the heart tissue of aging mice that received RSV treatment. RSV treatment also attenuated apoptosis, MMP of cardiomyocytes, and ROS content in aging mouse cardiomyocytes, suggesting that RSV exerted cardioprotective effects by inhibiting apoptosis.

Inflammation plays a significant role in the pathogenesis of CVD. RSV has been reported to exert multiple biological activities against inflammation, oxidative stress, and aging (Li et al., 2018). Our results demonstrated that in the D‐gal‐induced group, levels of IL‐1β, TNF‐α, TLR4, NF‐κB p65, and apoptotic responses were significantly enhanced. Furthermore, RSV markedly alleviated these phenomena through the NF‐κB pathway, suggesting that RSV could regulate inflammation and promote cardiac health in aging mice. Recently, it has been reported that Notch and NF‐κB played a key role in cell–cell contact for activation to regulate cell fate decisions. This constituted a new exciting field to illustrate multiple complex mechanisms referring to the cardioprotection of RSV (Osipo et al., 2008). In many physiological processes, such as cell growth, proliferation, differentiation, and organ development, activation of the Notch signaling pathway is critical (Samarajeewa et al., 2019). Furthermore, it was involved in various pathological processes, such as oxidative stress and inflammation, as well as cardiovascular development and differentiation (Nistri et al., 2017). Mammal Notch receptors were classified as Notch 1–4, together with five transmembrane ligands (Jagged1, Jagged2, Delta‐like1, Delta‐like3, and Delta‐like4; Borggrefe & Oswald, 2009). Studies have shown that the intracellular segment gene (NICD) of the Notch 1 receptor transfected with adenovirus after myocardial infarction may improve hemodynamics, suggesting that Notch gene re‐expression was an adaptive response secondary to myocardial injury and is closely associated with the repair and regeneration of viable myocardium (Kasahara et al., 2013). Notch also plays a cardioprotective role by upregulating Hes‐1 (Fang et al., 2020). Reports have shown that almost all subtypes of Notch receptor and ligand were upregulated at different levels during the remodeling of myocardium that was imparted (Anbara et al., 2020). Notch is connected with the NF‐κB signaling pathway at various levels. For instance, NF‐κB upregulated the transcription of one of the ligands of the Notch receptor (Jagged‐1; Moore et al., 2020). Additionally, NF‐κB stimulated the expression of hes‐5 and Deltex‐1, both of which were targets of Notch (Osipo et al., 2008). Our results demonstrated a significant increase in the expression of Notch 1 protein in the D‐galactose‐induced group, which was significantly reduced by RSV treatment. Therefore, RSV may exert its cardiac protective effects by activating the Notch/NF‐κB signaling pathway.

Currently, the relationship between gut microbes and aging has been attracting increased interest from researchers. SCFAs, metabolites of intestinal microorganisms, are important physiologically functional substances in the intestinal tract. Acetate, which is a predominant SCFA found in both the gut and systemic circulation, has been observed to attenuate CVD (George et al., 2020). In addition, a possible mechanism underlying the anti‐inflammatory effects of SCFAs is their utilization as metabolic substrates. In comparison to lipids, SCFAs were capable of bypassing multiple β‐oxidation cycles prior to their entry into the tricarboxylic acid cycle, thereby reducing oxygen consumption and the production of ROS (Evans et al., 2020). ROS accumulation is positively associated with inflammation (Rizwan et al., 2014). Our results showed that SCFAs were notably reduced in D‐gal‐induced aging mice, while SCFA species and content escalated after RSV treatment. Strikingly, we found that RSV could improve the level of intestinal SCFAs in the D‐gal‐induced aging mice. Moreover, the present study also uncovered that RSV can reduce intestinal pH, which is facilitative to the growth and proliferation of probiotics and thereby has a protective effect on the body's aging process. Therefore, our findings suggested that the cardioprotective effects of RSV were linked to its role in increasing the level of intestinal SCFAs and decreasing intestinal pH. However, direct evidence of this gut–heart crosstalk has not been confirmed in the present study since we did not investigate the gut microbiota community and metabolites.

## 5 CONCLUSION

Overall, our data indicated that RSV protected against age‐related heart impairment by reducing histopathological changes, attenuating apoptosis, and maintaining redox balance in the heart tissue. Notably, RSV‐induced cardioprotection appeared to involve repairing aberrant inflammatory responses through crosstalk between the Notch/NF‐κB pathways in D‐gal‐induced aging mice. Furthermore, RSV significantly increased gut microbial metabolite SCFAs and modulated pH values in feces, suggesting that its role in intestinal protection may also contribute to its protective effects against age‐related heart injury in vivo.

## AUTHOR CONTRIBUTIONS

**Le‐feng Wang:** Conceptualization (equal); investigation (equal); methodology (equal); project administration (equal); visualization (equal); writing – original draft (equal); writing – review and editing (equal). **Wen‐Juan Li:** Funding acquisition (equal); writing – original draft (equal); writing – review and editing (equal). **Xian‐Yi Zhang:** Data curation (equal). **Yi‐Chi Zhang:** Methodology (equal). **Guang‐Feng Chen:** Visualization (equal). **Xing‐Yu Zhou:** Investigation (equal). **Dong‐Mei Xv:** Visualization (equal). **Qiong Wu:** Conceptualization (equal); project administration (equal); supervision (equal); writing – original draft (equal); writing – review and editing (equal).

## CONFLICT OF INTEREST STATEMENT

The authors have no potential conflicts of interest.

## Data Availability

The data that support the findings of this study are available on request from the corresponding author. The data are not publicly available due to privacy or ethical restrictions.
